# Metformin upregulates circadian gene PER2 to inhibit growth and enhance the sensitivity of glioblastoma cell lines to radiotherapy via SIRT2/G6PD pathway

**DOI:** 10.3389/fphar.2025.1563865

**Published:** 2025-03-17

**Authors:** Hailiang Li, Zheng Ma, Wanfu Yang, Yifan Zhang, Jinping Sun, Haifeng Jiang, Faxuan Wang, Li Hou, Hechun Xia

**Affiliations:** ^1^ Department of Radiation Oncology, General Hospital of Ningxia Medical University, Yinchuan, Ningxia, China; ^2^ Key Laboratory of Craniocerebral Diseases, Ningxia Medical University, Yinchuan, China; ^3^ Department of Otolaryngology, Head and Neck Surgery, General Hospital of Ningxia Medical University, YinChuan, Ningxia, China; ^4^ Department of Neurosurgery, General Hospital of Ningxia Medical University, Yinchuan, Ningxia, China; ^5^ Department of Pathology, General Hospital of Ningxia Medical University, Yinchuan, Ningxia, China; ^6^ School of Public Health and Management, Ningxia Medical University, Yinchuan, Ningxia, China

**Keywords:** glioblastoma multiform (GBM), metformin, period circadian regulator 2 (PER2), silent information regulator 2 (SIRT2), glucose-6-phosphate dehydrogenase (G6PD), pentose phosphate pathway (PPP)

## Abstract

**Introduction:**

Glioblastoma multiform (GBM) is considered the deadliest brain cancer. Standard therapies are followed by poor patient’s survival outcomes, so novel and more efficacious therapeutic strategies are imperative to tackle this scourge. Metformin has been reported to have anti-cancer effects. However, the precise mechanism underlying these effects remains elusive. A better understanding of its underlying mechanism will inform future experimental designs exploring metformin as a potential adjuvant therapy for GBM. This research aimed to elucidate the potential molecular mechanism of metformin in GBM by integrating proteomics and transcriptomics.

**Methods:**

The study examined the effects of metformin on GBM cell lines using various methods. The U87, U251 and HA1800 were cultured and modified through PER2 knockdown and overexpression. Cell viability was assessed using the CCK8 assay, and G6PDH activity and intracellular NADPH^+^ levels were measured with specific kits. ROS levels, mitochondrial membrane potential, cell cycle distribution and apoptosis were analyzed by flow cytometry. RNA was extracted for transcriptomic analysis through RNA sequencing, while proteomic analysis was performed on total protein from treated cells. WB detected specific proteins, and RT-qPCR quantified gene expression. In vivo experiments, GBM xenograft on nude mice treated with metformin combining radiotherapy was evaluated and received IHC and TUNEL staining for protein expression and apoptosis assessment. Statistical analyses were conducted using Prism software to identify significant group differences.

**Results:**

We found that differential expressional genes and proteins relating to circadian rhythm were enriched in proteomic or transcriptomic. The expression of PER2, the key circadian gene, was up-regulated in GBM cell lines when treated with metformin. Furthermore, the expression of silent information regulator 2(SIRT2) was down-regulated, while the expression of the G6PD protein just slightly increased in GBM cell lines. Meanwhile, NADPH+ production and G6PDH enzyme activity significantly decreased. Further study validated that metformin inhibited the cell growth of GBM cell lines through up-regulating *PER2* and inhibited SIRT2/G6PD signaling pathway, enhancing radiotherapy(RT) sensitivity. We also found that the inhibition of SIRT2 caused by metformin is mediated by PER2.

**Discussion:**

We found the pivotal role of metformin as an effective circadian rhythm regulator. Targeting circadian clock gene to modify and rescue the dysfunctional circadian clock of GBM cells at molecular level might be an innovative way to administer cancer chronotherapy and maintain metabolic homeostasis in real world practice.

## 1 Introduction

Glioblastoma multiforme (GBM) is the most common intrinsic malignant tumor of the central nerve system in adults, especially in the brain, with an incidence of 6 cases per 100,000 population per year (Shah, 2023). GBM is highly invasive and life-threatening, and the median overall survival for these patients is very poor at approximately 14.6 months, with a 5-year overall survival rate of about merely 4.9% (Agata et al., 2023). Though some improvements have been gained in maximum range tumor resection under navigation of functional magnetic resonance imaging during surgery, adjuvant radio-chemotherapy post-operation, adjuvant chemotherapy, tumor electric field therapy, and targeted therapy (Huang et al., 2021). Hence, a novel approach is desired for these GBM patients.

**TABLE 1:** Sequences of the primer.

Gene	Forward (5′-3′)	Reverse (3′-5′)
*PER2*	CAG​GTG​AAA​GCC​AAT​GAA​GA	GGG​AGG​TGA​AAC​TGT​GGA​AC
*G6PD*	TCA​GAG​GTG​CAG​GCC​AAC​AA	CAT​AGA​GGA​CGA​CGG​CTG​CA
*SIRT2*	CGC​ACG​GCA​CCT​TCT​ACA​CAT​C	GGC​TCT​GAC​AGT​CTT​CAC​ACT​TGG
*GAPDH*	CAG​GAG​GCA​TTG​CTG​ATG​AT	GAAGGCTGGGGCTCATTT

Drug repurposing is a strategy that utilizing current approved drugs for a novel alternative indication (Ashburn and Thor, 2004), which often reducing costs on drug development and the time to clinical translation (Pushpakom et al., 2018). Metformin, an oral biguanide derived from the French lilac, is the first-line and widely prescribed drug for type 2 diabetes mellitus patients (Benoit et al., 2012). Many studies have demonstrated encouraging anti-proliferative effect *in vitro* and could reduce tumor weight and volume in animal models (Benoit et al., 2012; Ng et al., 2020; He et al., 2020; Chen et al., 2021; Lv and Guo, 2020; Xiong ZS et al., 2019; Gallez et al., 2024). However, some researchers pointed that metformin might not be beneficial for recurrent or refractory GBM (Yoon et al., 2023).

According to the current literature, metformin has been proposed to have direct and indirect effects on cancer cells involving the activation of AMPK (Wang et al., 2019), and there have been many reports on this subject. For example, metformin has been reported to have a dual effect on breast cancer patients (Cejuela et al., 2022). The use of metformin has been linked to improved overall survival and progression-free survival in high-grade gliomas patients. Metformin can relieve brain edema and alter the microenvironment of brain tumors through reducing vascular permeability and mitigating edema-associated symptoms (Seliger et al., 2018). The adjuvant therapy of metformin in high-grade glioblastoma has been associated with improved outcomes, particularly in patients with MGMT promoter methylation. The synergistic effects of metformin with standard therapies could pave the way for its utility into GBM treatment. However, the precise mechanism underlying these effects remains elusive. Thus, a better understanding of metformin’s anti-tumor activity would help to optimize its clinical use for the benefit of GBM patients. In this study, we found that metformin can inhibit the cell growth and prompt death in U87/U251 GBM cell lines. Metformin upregulated and rescued period circadian regulator 2 (*PER2*) in GBM cell lines, inhibited their growth by inhibiting the silent information regulator 2 (SIRT2)/glucose-6-phosphate dehydrogenase (G6PD) signaling pathway, and hence enhanced their radio sensitivity. The inhibition of SIRT2 induced by metformin is mediated by the *PER2* circadian gene. Here, we studied the effect of metformin and PER2 on the pentose phosphate pathway (PPP) and postulated that the strategy of using metformin to modify the circadian clock may provide an innovative way to administer chronotherapy for cancer treatment.

## 2 Results

### 2.1 Metformin inhibited the cell growth, proliferation and promoted apoptosis in GBM cell lines *in vitro*

**FIGURE 1:**
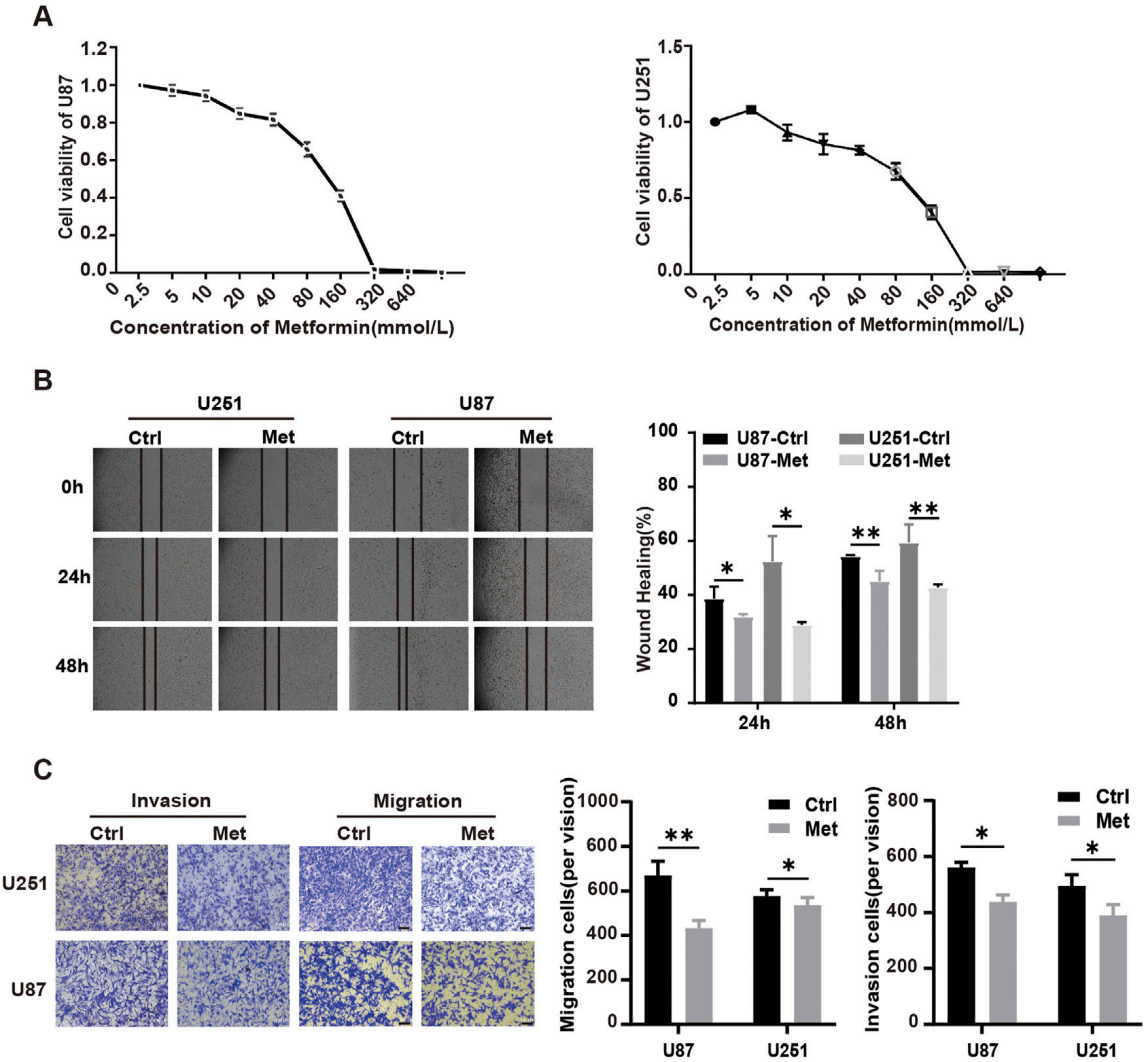
Metformin inhibited cell growth, proliferation, migration and invasion in GBM cell lines *in vitro*. **(A)** The effect of metformin on cell viability detected by the CCK8 assay in U87 and U251 cell lines. **(B)** Scratch test of U87 and U251 cell lines treated with metformin at 24h and 48h, and quantified by the healing rate. **(C)** Transwell assays to analyze the effect of metformin on the migration and invasion abilities of U87 and U251 (magnification, ×100), and quantified by the cell number. Data are expressed as the means ± standard deviation (SD). *P < 0.05, **P < 0.01, ***P < 0.001. Ctrl: control, Met: metformin.

To explore the role of metformin in GBM cell lines, CCK8 was used to screen the optimal action concentration of metformin in glioma cells using escalating concentrations of 5, 10, 20, 40, 80, 160, 320, and 640 mmol/L *in vitro*. The results showed that the cell vitality of U87 and U251 glioma cell lines gradually decreased, and the cell inhibition rate gradually increased inversely with the increase in metformin concentration (Figure 1A). The optimal metformin concentration to inhibit glioma cell lines was 20 mmol/L according to the CCK8 assay. Next, we used 20 mmol/L of metformin in the following study. The scratch test and migration test were used to detect the effect of metformin on cell migration in the U87 and U251 cell lines. Compared with the control group, the scratch healing rate of the metformin treatment group significantly decreased at 24h and 48h (*P* < 0.05) (Figure 1B). The transwell migration test showed that the number of cells that migrated in the metformin treatment groups of the U87 and U251 cell lines significantly decreased compared with the control group. Similarly, the number of cells that migrated through the chamber treated with metformin in the U87 and U251 cell lines significantly decreased in the transwell invasion test (*P* < 0.05) (Figure 1C).

**FIGURE 2:**
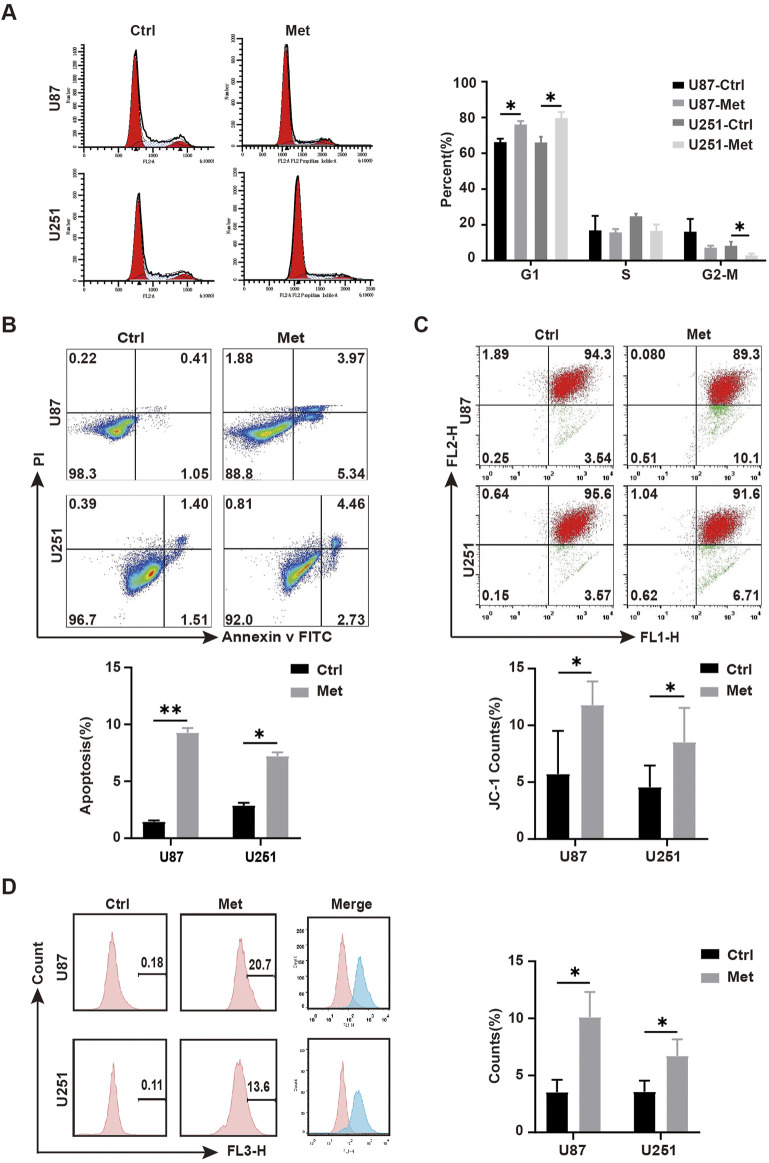
Metformin promoted apoptosis in GBM cell lines *in vitro*. **(A)** Cell cycle distribution detected by flow cytometry after metformin treatment at 24h, and quantified by the cell distribution rates. **(B)** Cell apoptosis detected by flow cytometry in metformin treatment and control groups at 24h, as quantified by the apoptosis rate. **(C)** JC-1 was detected by flow cytometry in metformin treatment and control groups at 24h, and quantified by the apoptosis rate. **(D)** ROS was detected by flow cytometry in the metformin treatment and control groups at 24h, and quantified by the apoptosis rate. Data are expressed as the means ± standard deviation (SD). *P < 0.05, **P < 0.01, ***P < 0.001. Ctrl: control, Met: metformin.

Flow cytometry was performed to analyze the influence of metformin on the cell cycle distribution and apoptosis. The results showed that compared with the control groups, the metformin treatment group had a significantly higher proportion of U87 and U251 GBM cell lines arrested in G1 phase (*P* < 0.05) (Figure 2A). The apoptosis rates of U87 and U251 cells in the metformin treatment group significantly increased compared with the control group (Figure 2B). Further detection of early apoptosis using JC-1 showed that the apoptosis rate significantly increased (*P* < 0.05) (Figure 2C), and the intracellular reactive oxygen species (ROS) production increased in the metformin treatment group (*P* < 0.05) (Figure 2D). These results suggested that metformin inhibited cell growth, proliferation, and migration, and promoted apoptosis in GBM cell lines U87 and U251.

### 2.2 Metformin upregulated PER2, downregulated SIRT2, and inhibited the enzyme activity of G6PD(H) in GBM cell lines

**FIGURE 3:**
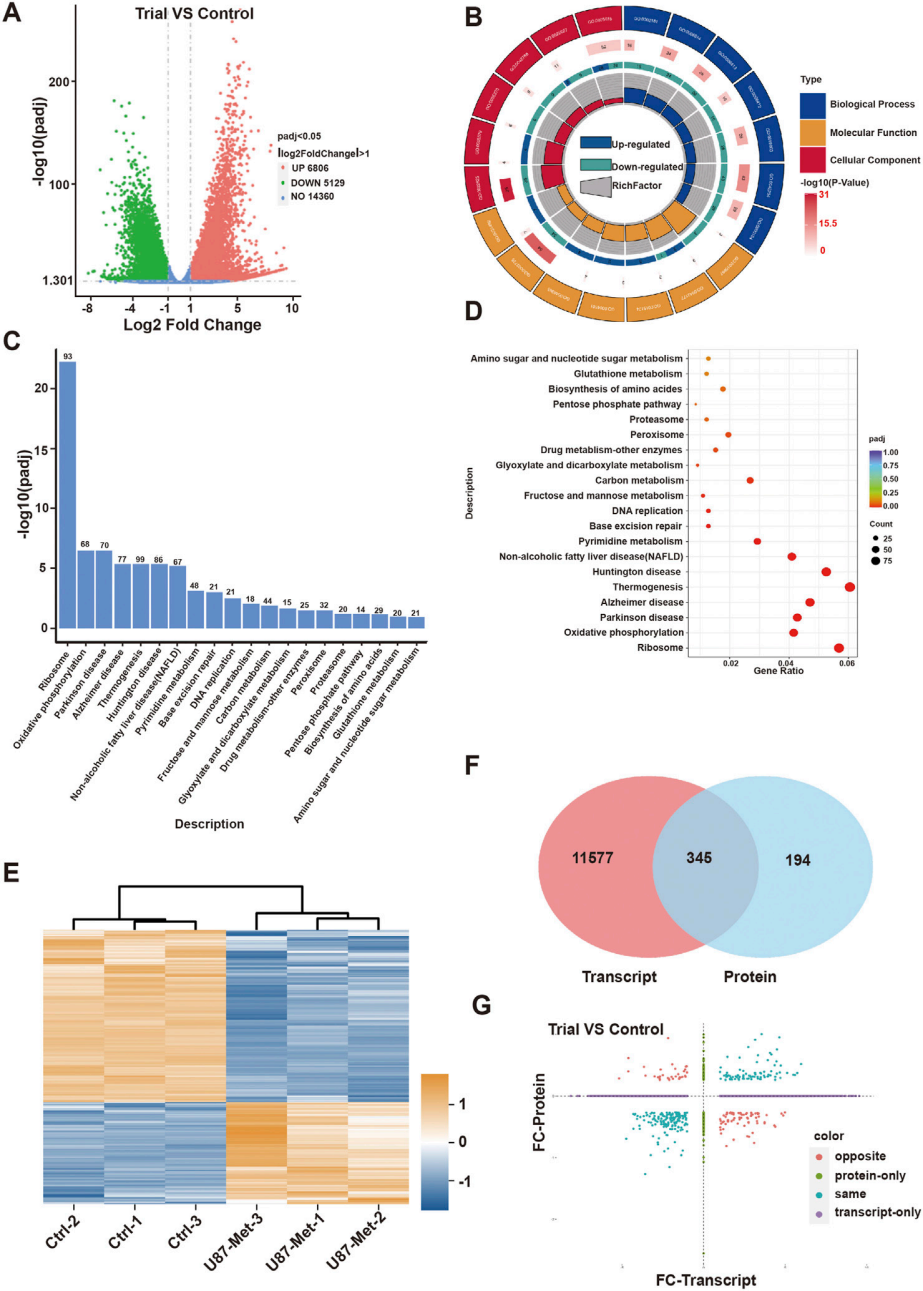
The mechanism of action of metformin in GBM cell lines. **(A)** Volcanic map of differentially expressed genes(DEGs) in transcriptomic. **(B)** GO enrichment of DEGs in transcriptomic. **(C)** KEGG enrichment pathway of DEGs in transcriptomic. **(D)** GO enrichment of biological process in of DEGs in transcriptomic. **(E)** Heat map of differentially expressed proteins in proteomic. **(F, G)** Integrative analysis was conducted on the proteomic and transcriptomic data.

We next explored the mechanism involved in the anti-tumor effect of metformin in GBM cell lines. First, transcriptomic detection was performed on U87 cell lines treated with/without metformin. The results showed that there were 11,935 differentially expressed genes (DEGs), of which 6,806 were upregulated and 5,129 were downregulated (Figure 3A). Gene Ontology (GO) enrichment analysis and CIRCOS image containing molecular function, biological process, and cellular component was seen in Figure 3B. The top 20 signaling pathways in the comprehensive Kyoto Encyclopedia of Genes and Genomes (KEGG) includes PPP signaling pathway, which containing 14 of the DEGs is closely related to cell proliferation (Figures 3C, D; Supplementary Fgiure S1; Supplementary Table S1) and G6PD was included in the 14 DEGs (Supplementary Table S1). G6PD is the key rate-limiting enzyme in the PPP signaling pathway, and SIRT2 is essential for the active dimer of G6PD through deacetylating G6PD protein at lysine 403 (Xu et al., 2016; Ye et al., 2016). Proteomic results showed that there were 539 differential expression protein, of which 200 were upregulated and 339 were downregulated (Figure 3E). Then, integrative analysis was conducted on the proteomic and transcriptomic data. The results showed 345 DEGs at both transcriptional and protein levels, 11,577 DEGs at only the transcriptional level, and 194 only at the protein level (Figures 3F, G).

**FIGURE 4:**
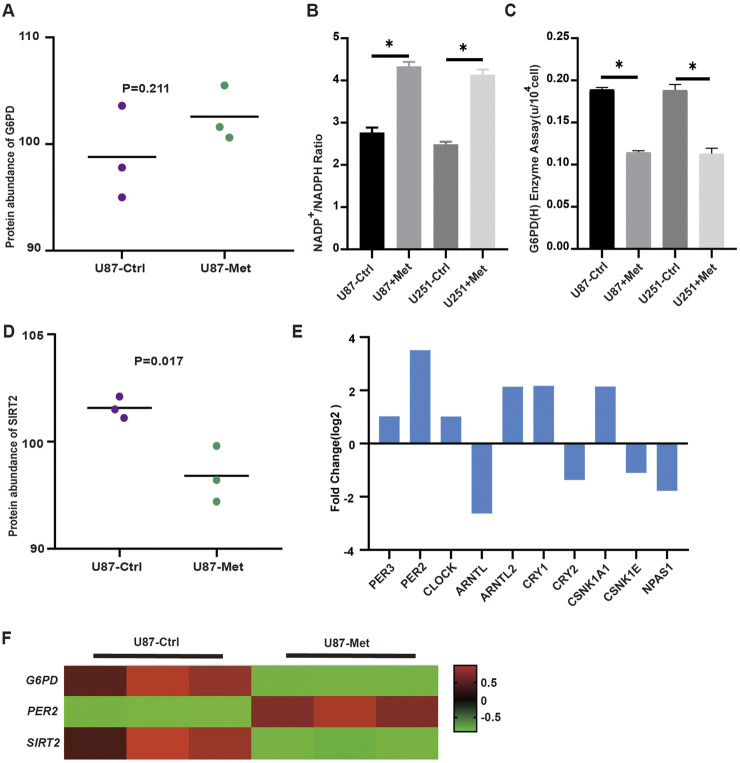
The expression of PER2, G6PD and SIRT2 in proteomic and transcriptomic. **(A)** The expression levels of G6PD were analyzed in proteomic. **(B)** NADPH production in U87 and U251 cells was measured before and after metformin treatment. **(C)** The activity of G6PDH in U87 and U251 cells was measured with and without metformin treatment. **(D)** The expression level of SIRT2 were analyzed in proteomic treated with metformin. **(E)** The expression level of circadian gene in transcriptomic. **(F)** The transcriptomic data showed that *PER2*, *G6PD* and *SIRT2* expression in GBM cell lines after metformin treatment. Data are expressed as the means ± SD. *P < 0.05, **P < 0.01, ***P < 0.001.

We further estimated the expression of G6PD proteins according to the proteomic data. The G6PD protein expression level did not significantly change (Figure 4A). As is known to us, G6PD protein acts as the first rate-limiting and the primary enzyme in the PPP pathway, so NADPH^+^ production reflects the flux of this pathway. Thus, we detected NADPH^+^ production in U87 and U251 GBM cell lines treated with metformin to classify its effect on the pathway. The results showed that NADPH^+^ production decreased significantly in GBM cell lines when treated with metformin (Figure 4B), suggesting that this drug might inhibit the PPP pathway. But we found that G6PD protein expression level did not decline but increased slightly in U87 and U251 cell lines when treated with metformin. This meant that metformin might not inhibit G6PD protein expression. Did metformin depress the G6PD enzyme activity in the PPP? Or was G6PD protein inactivated in this situation? Thus, G6PDH enzyme activity was measured in GBM cell lines with and without metformin intervention. The results showed that G6PDH enzyme activity decreased significantly after the metformin intervention (Figure 4C). It suggested that metformin affected the enzyme activity of G6PDH. Since G6PDH enzymatic activity is mainly regulated by the deacetylase SIRT2, SIRT2 expression after metformin was investigated. As is expected, the following results revealed that SIRT2 expression level in GBM cell lines decreased significantly after metformin treatment (Figures 4D, F). Several studies have shown that SIRTs is regulated by biorhythm (Rodríguez-Santana et al., 2023; Sun et al., 2019). In our study, transcriptomic analysis showed that up to 10 of core biorhythm gene changed significantly after metformin treatment, especially the upregulation of *PER2* (Figure 4E; Supplementary Figure S2; Supplementary Table S2). The transcriptomic data also showed that *PER2* was upregulated, while *G6PD* and *SIRT2* expression was downregulated in GBM cell lines after metformin treatment (Figure 4F), which sparked our intense interest to investigate what happened to G6PD, SIRT2, PER2, and metformin in the PPP signaling pathway.

**FIGURE 5:**
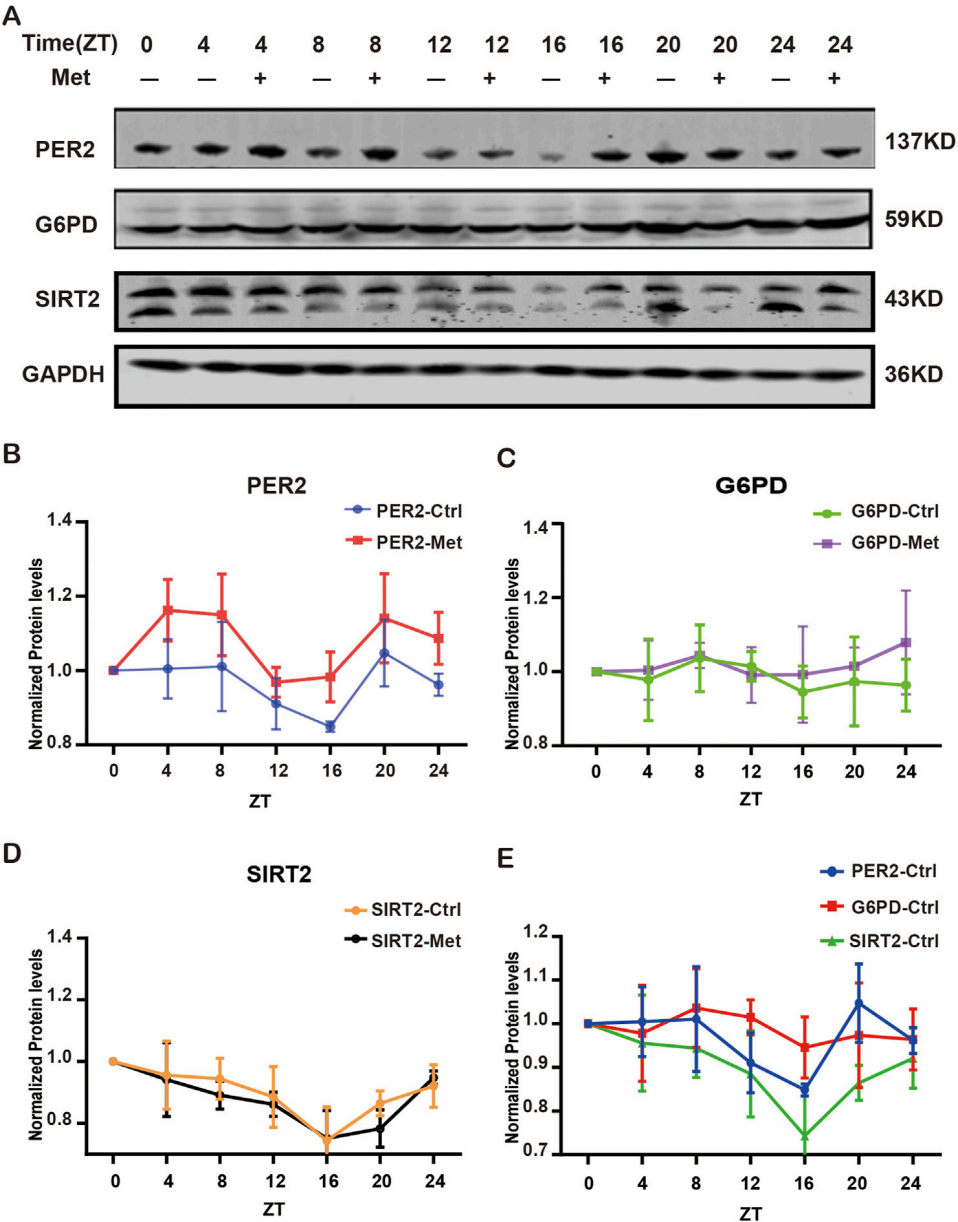
The expression of G6PD, SIRT2 and PER2 proteins treated with/without metformin. **(A)** Western blotting analysis of the expression of G6PD, SIRT2 and PER2 proteins over 24h with/without metformin treatment. **(B)** Densitometric quantification of PER2 proteins in 24h with/without metformin treatment. **(C)** Densitometric quantification of G6PD proteins in 24h with/without metformin treatment. **(D)** Densitometric quantification of SIRT2 proteins in 24h with/without metformin treatment. **(E)** Densitometric quantification of PER2, G6PD and SIRT2 proteins in 24h without metformin. Ctrl, control; Met, metformin.

To further validate the expression levels of G6PD, SIRT2, PER2 in GBM cell lines and explore the interaction among G6PD, SIRT2, PER2, and metformin, we detected the expression of these indicators dynamically during 24h in the condition of metformin intervention or without (Figure 5A). The results presented the rhythmic expression of PER2. After treatment with metformin, the PER2 rhythms did not change, but the amplitude of PER2 increased significantly (Figure 5B). we were pleasantly and surprised to find that G6PD and SIRT2 also exhibited rhythmic expression, besides that the amplitude of SIRT2 decreased significantly, but there was no significant change in G6PD (Figures 5C, D). Moreover, the rhythms of G6PD and SIRT2 were similar to that of PER2’s (Figure 5E). Does this phenotype mean that the rhythms of G6PD and SIRT2 is modulated by PER2?

**FIGURE 6:**
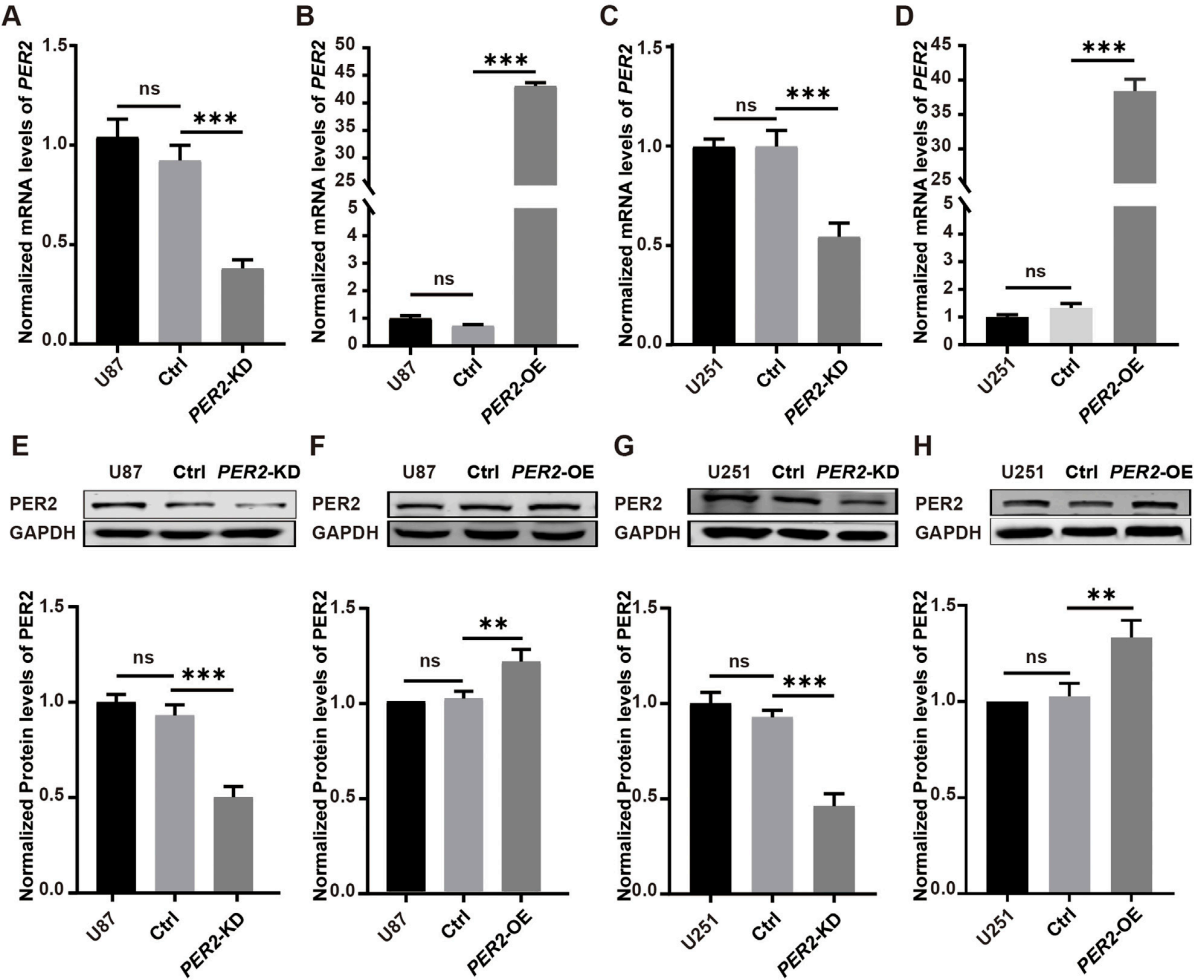
Verify *PER2*-KD and *PER2*-OE cell models of the U87 and U251. **(A–D)** The relative mRNA levels of *PER2* in U87-*PER2*-KD, U87-*PER2*-OE, U251-*PER2*-KD and U251-*PER2*-OE cells. **(E–H)** Western blotting analysis and densitometric quantification of the expression of PER2 proteins in U87-*PER2*-KD, U87-*PER2*-OE, U251-*PER2*-KD and U251-*PER2-*OE cells. Data are expressed as the means ± SD. *P < 0.05, **P < 0.01, ***P < 0.001. KD, knock down; OE, overexpression.

Next, lentivirus was used to construct and verify *PER2-*knockdown (*PER2*-KD) and *PER2*-overexpression (*PER2*-OE) cell models of the U87 and U251 (Figure 6).

**FIGURE 7:**
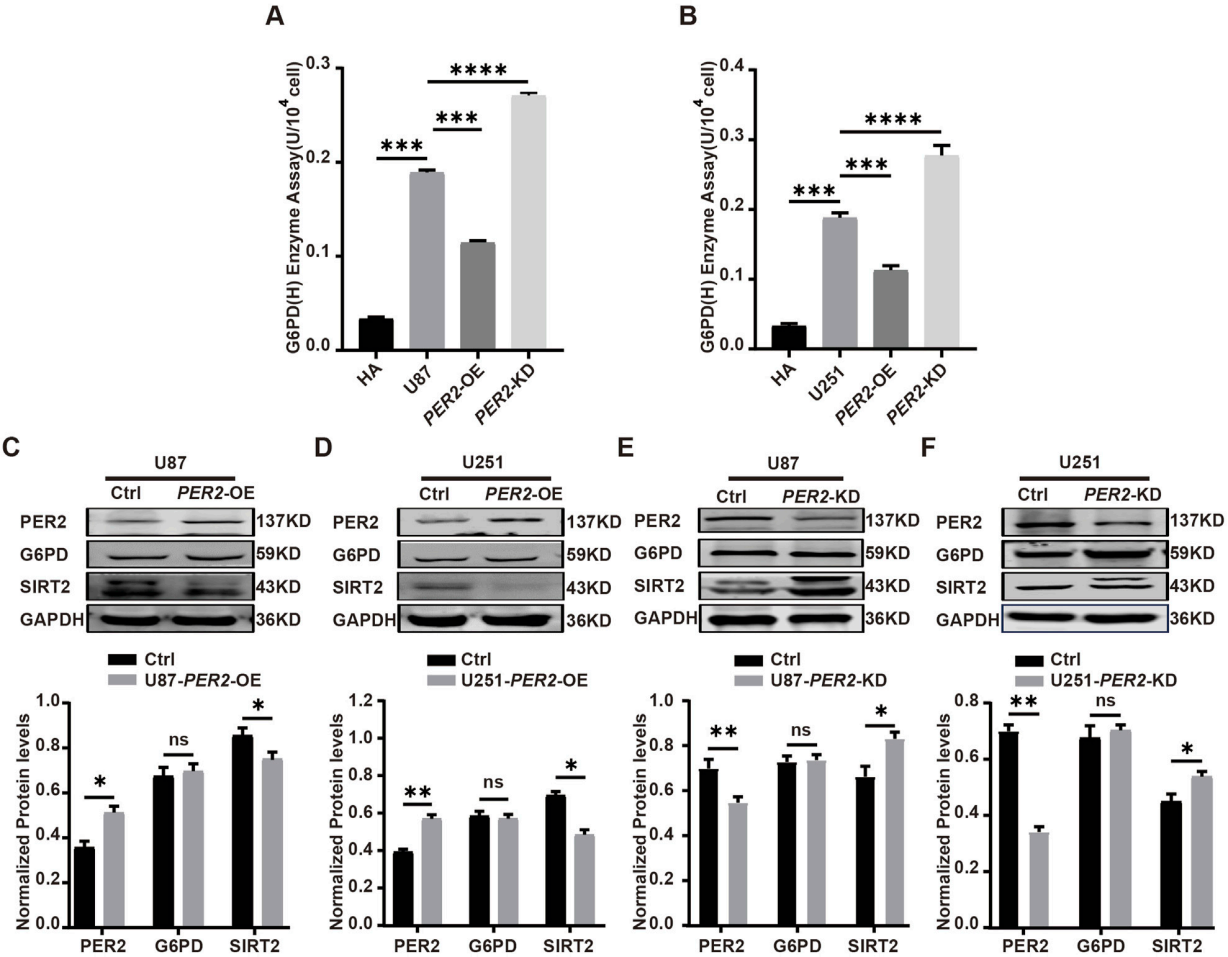
Validate the expression level of G6PD, SIRT2, PER2 in GBM cell lines. **(A)** The activity of G6PDH was measured in U87-*PER2*-KD, U87- *PER2*-OE cells. **(B)** The activity of G6PDH was measured in U251-*PER2*-KD, U251- *PER2*-OE cells. **(C)** Western blotting analysis of the expression of G6PD and SIRT2 proteins in U87- *PER2*-OE cells. **(D)** Western blotting analysis of the expression of G6PD and SIRT2 proteins in U87- *PER2*-OE cells. **(E)** Western blotting analysis of the expression of G6PD and SIRT2 proteins in U251-*PER2*-KD cells. **(F)** Western blotting analysis of the expression of G6PD and SIRT2 proteins in U251-*PER2-*OE cells. Data are expressed as the means ± SD. *P < 0.05, **P < 0.01, ***P < 0.001. KD: knock down, OE: overexpression.

Then, G6PDH enzyme activity and G6PD protein expression level were detected in each model. The results showed that G6PDH enzyme activity decreased significantly in *PER2*-OE GBM cell lines and increased significantly in *PER2*-KD GBM cell lines (Figures 7A, B). The SIRT2 protein decreased significantly in *PER2*-OE GBM cell lines and increased significantly in *PER2-*KD cell lines, a same trend with G6PDH. While, the G6PD protein changed slightly in these GBM cell line models (Figures 7C–F). These results suggested that PER2 might affect the protein expression of SIRT2 while affecting the enzymatic activity of G6PDH.

**FIGURE 8:**
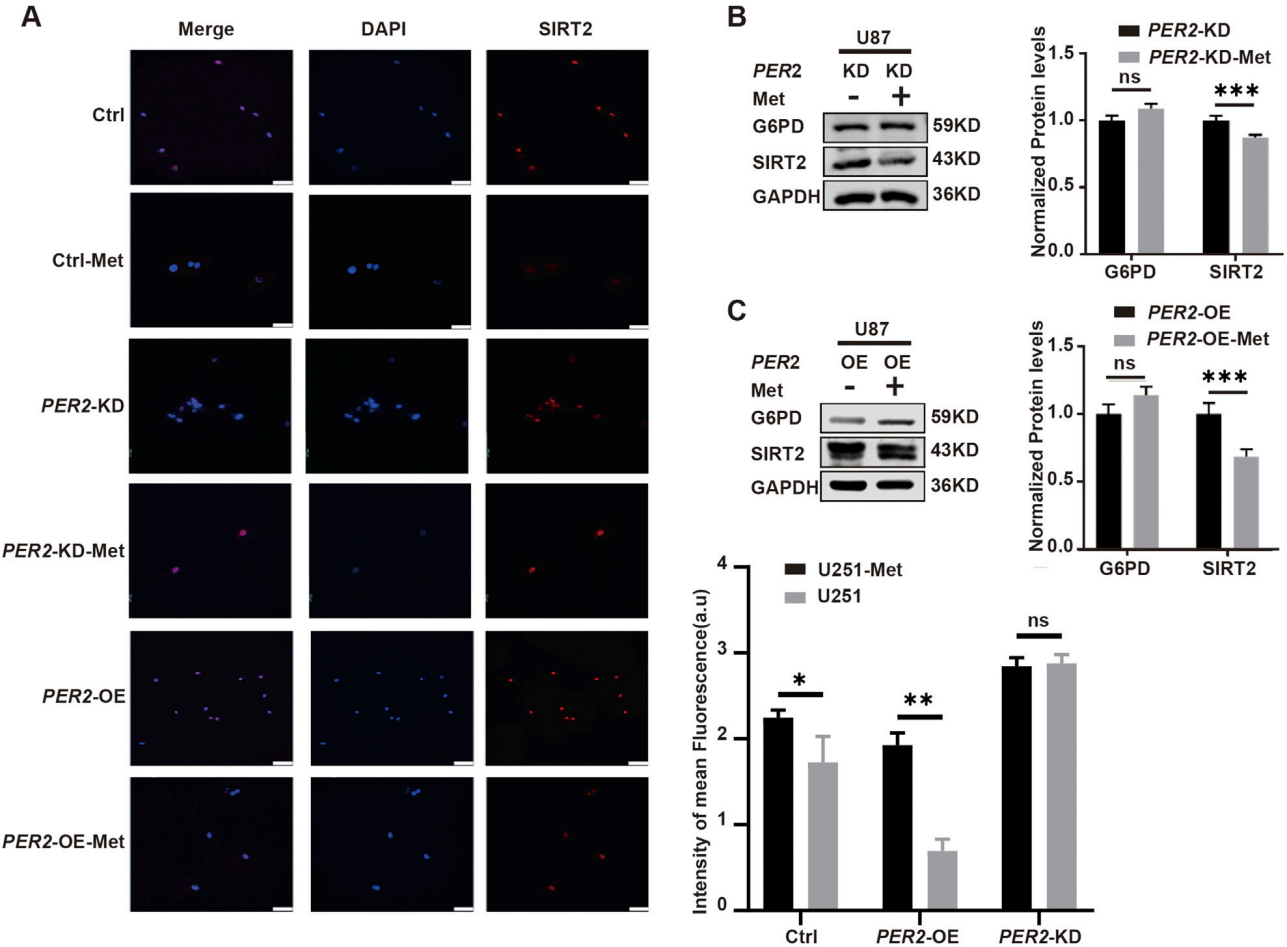
Clarify the influence of metformin on PER2 and SIRT2. **(A)** Representative fluorescence and densitometric quantification intensity of SIRT2 in Ctrl, U251-*PER2*-KD, U251-*PER2*-OE cells treated with/without metformin. **(B)**. Western blotting analysis of the expression of G6PD and SIRT2 proteins in U87-*PER2*-KD GBM cells treated with/without metformin. **(C)**. Western blotting analysis of the expression of G6PD and SIRT2 proteins in U87-*PER2*-OE GBM cells treated with/without metformin. Data are expressed as the means ± SD. *P < 0.05, **P < 0.01, ***P < 0.001. KD: knock down, OE: overexpression, Ctrl: control, Met: metformin.

To further clarify the relationship among metformin, PER2 and SIRT2, we treated *PER2*-OE and *PER2*-KD U87 cell lines with metformin. The results showed that SIRT2 expression significantly decreased in *PER2*-OE U87 cell lines but slightly decreased in *PER2*-KD cell lines (Figures 8B, C). Immunofluorescence showed the same results (Figure 8A). These results suggest that the inhibition of SIRT2 caused by metformin is mediated by PER2.

### 2.3 Metformin inhibits the growth of GBM xenograft tumors in nude mice via the PER2/SIRT2/G6PD signaling pathway and enhances radiotherapy sensitivity

**FIGURE 9:**
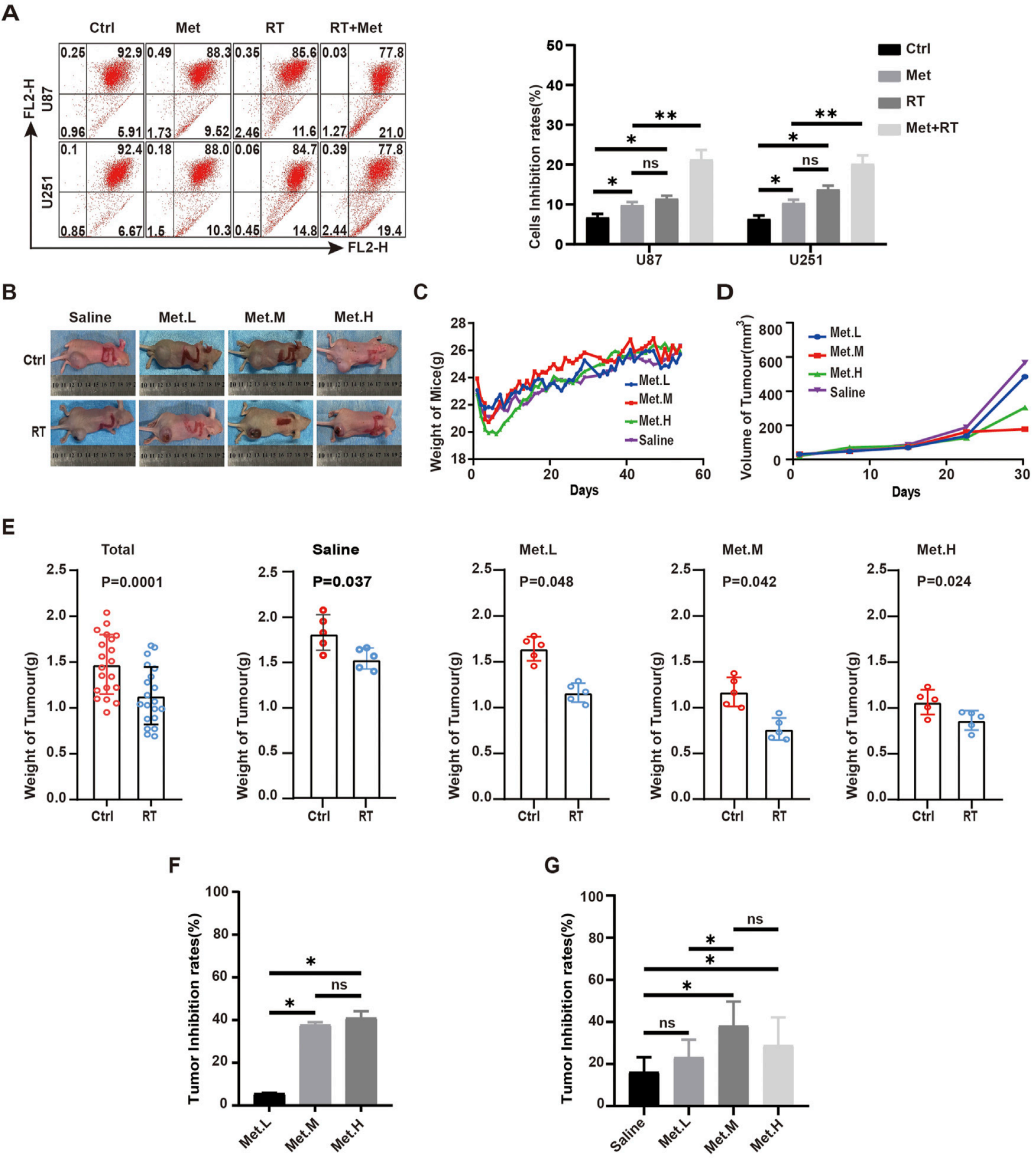
Metformin inhibits the growth of GBM xenograft tumors in nude mice. **(A)** In GBM cells, JC-1 were detected by flow cytometry in the metformin treatment group and RT groups at 24 h. **(B)** Photographs of mice and tumor tissues collected from the four groups at the end of treatment. **(C)** Body weights of mice during the treatment period. **(D)** Tumors volumes in the four groups during the therapeutic period. **(E)** Tumor weight in the four groups in control and radiotherapy group. **(F)** Tumor inhibition rates of in each control group according to tumor weight. **(G)** Tumor inhibition rates of in each control and RT group according to tumor weigh. Ctrl: control, Met: metformin. Data are expressed as the means ± SD. *P < 0.05, **P < 0.01, ***P < 0.001.

To study the anti-tumor effects of metformin during GBM radiotherapy, U87 and U251 cell lines treated with metformin for 24 h were exposed to 2Gy X-ray to detect JC-1 expression. The results show that JC-1 increased more in the group treated with radiotherapy and metformin than in the groups treated with radiotherapy alone or metformin alone (Q-value, 1.15) (Figure 9A). This suggests the synergistic effect of radiotherapy and metformin on GBM cell lines.

To further verify the effect and mechanism underlying the effects of metformin on GBM cell lines *in vivo*, 40 nude mice were used to construct subcutaneous xenograft tumor model with U87 cell lines and randomly divided into four groups (five in each group): group 1 (metformin, 100 mg/kg·d, Met. L), group 2 (metformin, 200 mg/kg·d, Met. M), group 3 (metformin, 300 mg/kg·d, Met. H), and a saline group as control (saline, 200 uL, Saline) (Figure 9B). The nude mice were given metformin or saline via gavage once a day from the day of xenograft tumor formation on. Nude mice body weight and tumor volume (length*width^2^)/2 were recorded after tumor formation. There was no significantly difference in the weight of nude mice among the experiment groups (Figure 9C). Tumor volume decreased significantly with the increased metformin dosage, and this trend was more obvious in the Met. L and Met. M groups (Figure 9D). When the transplanted xenograft tumor volume reached about 0.5cm^3^, the tumors in each group were divided into external beam of radiotherapy (RT) group and control group. When receiving RT, the xenograft tumors were given total dose of 18Gy of X-ray radiation with dose fraction of 6Gy across three fractions on an alternate day. After radiotherapy, anti-tumor effects among the groups were analyzed. The results shown that tumor weight receiving radiotherapy was significantly lower than that of the control group, and the mean xenograft tumor weight of the radiotherapy was lower than the control in each group (*P* < 0.05) (Figure 9E). In non-RT group, the analyses result showed the increasing tumor inhibition rate with the improvement of drug dosage, but there is no significant difference between Met. M and Met. H (Figure 9F). The analysis showed that there were static differences between RT and non-RT group and also among the different dose groups (Saline (16%), Met. L (23%), Met. M (38%), and Met. H (27%)) which received radiotherapy (Figure 9G). The combinational interaction of metformin and radiotherapy was further calculated, and corresponding Q-values were obtained: Met. L (1.39), Met. M (1.27), and Met. H (0.83). This suggested that low-dose of metformin (100 mg/kg/d) and medium-dose of 200 mg/kg/d had synergistic effects on the xenografts, while the high-dose metformin at 300 mg/kg/d (Met. H) had an antagonistic effect with radiotherapy.

**FIGURE 10:**
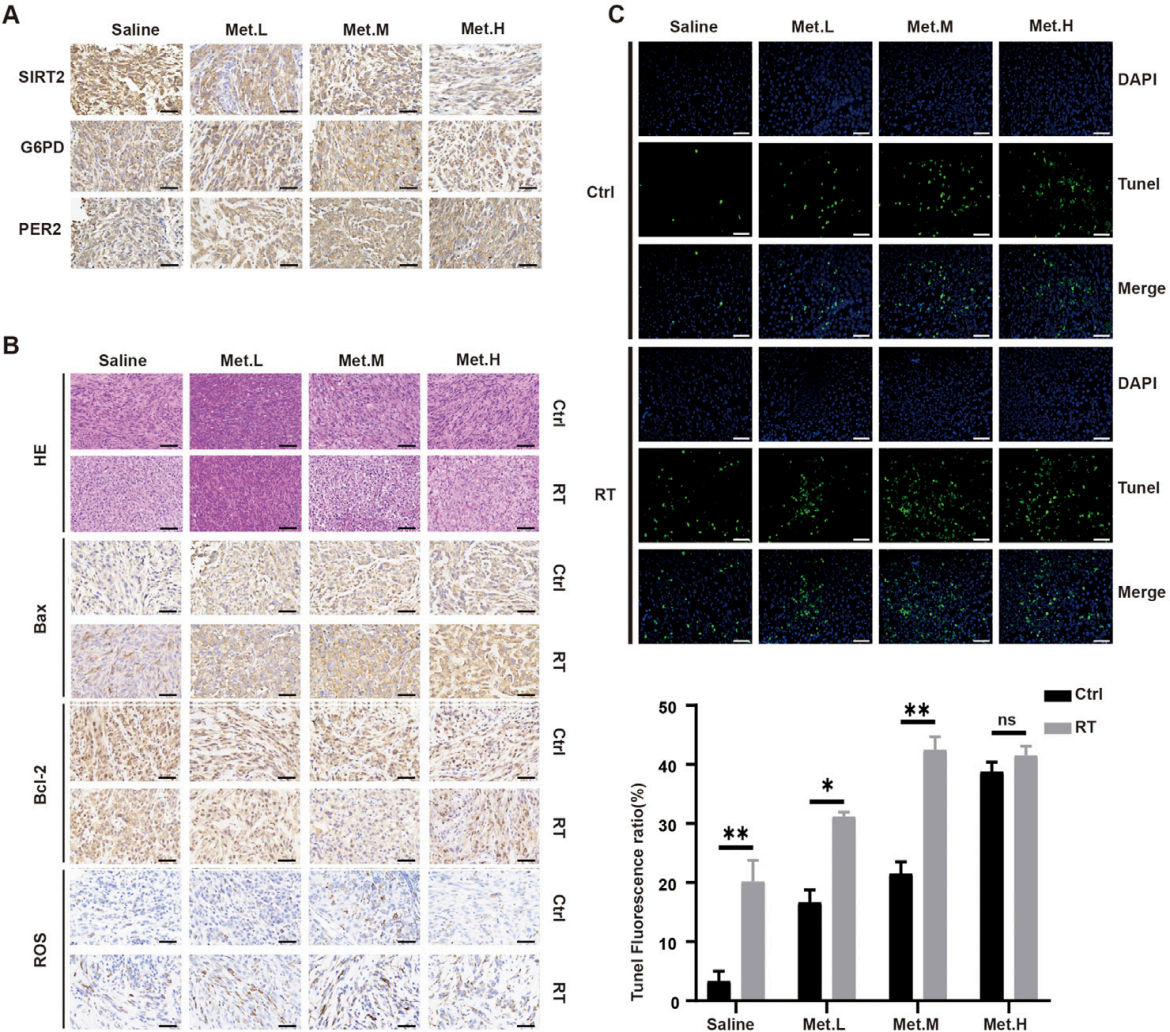
Detection the levels of proteins in the section GBM xenograft tumor in four groups. **(A)** IHC was used to analyze the expression levels of PER2, G6PD and SIRT2 proteins in the section GBM xenograft tumor in four groups. **(B)** H&E staining and Bax, Bcl-2, Ros staining of tumor sections after RT in the four groups. **(C)** Immunofluorescence was performed to TUNEL in the section GBM xenograft tumor, and densitometric quantification of fluorescence ratio of TUNEL in the section GBM xenograft tumor. These results are expressed as the means ± SD. *P < 0.05, **P < 0.01, ***P < 0.001. Magnification, ×400. Ctrl: control, Met: metformin.

The corresponding expression levels of PER2, G6PD, and SIRT2 in each group was detected via immunohistochemistry (IHC). The figure below revealed the increasing PER2 protein expression level in the Met. L, Met. M, and Met. H groups. However, there was no significant difference in the G6PD expression *in vivo*. SIRT2 expression in the tumor was significantly downregulated with the increased drug dosage (Figure 10A). Further detection of apoptosis-related markers showed that BAX increased after radiotherapy; ROS increased and Bcl-2 expression decreased in and between groups (Figure 10B). Remarkable differences of ROS and Bcl-2 expression were noted in the Met. M group (200 mg/kg/d). We also performed tunnel detection. Tunnel detection showed that the fluorescence in all radiotherapy groups significantly increased, and this was most prominent in the Met. M group (Figure 10C). Taken together, all these results suggest that metformin upregulates PER2, inhibits transplanted xenograft tumor growth in nude mice by inhibiting the SIRT2/G6PD signaling pathway and enhances radiotherapy sensitivity.

## 3 Discussion

In mammals, biorhythms or circadian rhythms are formed and influenced by many stimuluses such as the day–night cycle, temperature, and diet, etc. These are essential for the health of species (Lazar and Lazar, 2016). The intrinsic circadian clock determines the rhythmic features of almost all physiological and pathological processes, including blood pressure, heart rate, hormone levels, breathing, carcinogens, and many more (Wolf and Kramer, 2020). Many pathological events occur at specific times of the day; for example, cardiovascular and cerebrovascular events often happen at night (Sinturel et al., 2020). In mammals, the circadian clock system is a complex and multiple layered structure, containing the main clock housed in the suprachiasmatic nucleus (SCN) and the sub clocks of peripheral tissue and organs. There are 14 core central circadian genes and numerus clock-controlled genes. The core clock genes that control the central circadian network are Brain and Muscle Arnt-like protein (BMAL1) and the PERs gene family (Allada et al., 2021). These exist in multiple paralogs, the most prominent is BMAL1 and PER2. BMAL1 stimulates PER2 expression as an activator, and PER2 acts as a repressor, causing REV-ERBs to repress BMAL1 and resulting in approximately a 24 h daily rhythmic gene expression cycle (Mohawk et al., 2012). As mentioned above, the rhythmic onset can be influenced by the rhythmic expression of PER2. Actually, the circadian clock regulates biological cycles across species and is crucial for physiological activities and biochemical reactions, including oncogenesis, cancer onset and development (Shafi and Knudsen, 2019). The interplay between the circadian rhythm and cancer involves cell division, DNA repair, immune function, hormonal balance, and the treatment that is known as chronotherapy (Altman and biology, 2016; Fuhr et al., 2018; Lu et al., 2020; Koritala et al., 2021; Hou et al., 2020). This highlights the importance of maintaining a healthy circadian rhythm for cancer prevention and treatment.

Circadian misalignments are common and result in many diseases owing to artificial lighting, day–night shift work, jet lag, feeding time, etc., in modern society (Nam et al., 2023). Among these effects, day–night shift work is approved strongly associated with breast cancer (Gehlert et al., 2020). In pathological conditions, the biological rhythms of organs and tissues are disturbed. Previous studies of our team on GBM showed that multiple core clock genes disturbances occur in these patients, including PER2, BMAL1, and CLOCK. Some of these clock genes are related to the radiotherapy and chemotherapy sensitivity of GBM (Ma et al., 2020; Zhang et al., 2022; Niu et al., 2024; Xia et al., 2014; Yao et al., 2023; Zhanfeng et al., 2016). The response to medication is often affected by the time point that the medication was delivered. If the treatment is adapted to daily variations, it is likely to be more effective. In our research team, we found that GBM patients had a better outcome when received radiotherapy in the afternoon compared to receiving radiotherapy in the morning which is consistent with rhythmic peak of PER2 gene expression (Niu et al., 2024). Hence, comprehensive understanding of the underlying mechanism can help us to prevent and treat diseases.

As is mentioned above, circadian rhythm systems consist of central oscillators in the SCN of the brain and peripheral oscillators in almost all cells throughout the body. Biological clock systems are regulated through the transcription/translation feedback loops (TTFLs) of biorhythm genes. TTFLs include positive and negative feedbacks; for instance, the PER/CRY protein polymer is a negative feed-back that inhibits BMAL1/CLOCK heterodimer activity, which reversely inhibits PER/CRY and REV-ERBα gene transcription. The negative feedback loop refers to the REV/ERB polymer and the ROR reaction element of Bmal1 to control BMAL1/CLOCK expression (David et al., 1997; Patke et al., 2020). Through this feedback mechanism, biorhythms strictly control cell rhythm and maintain cell function. Studies have shown that the biorhythm system is crucial for glycogen synthesis in the liver, processing glucose in skeletal muscle, and thermogenic integration processes that guide glucose storage and utility in the energy cycle (Lazar and Lazar, 2016). This may be because the main output involves the rhythm control of enzymes involved in NAD^+^ biosynthesis. NAD^+^ is a cofactor in DNA repair pathways involving poly (ADP-ribose) polymerase (PARP) enzymes and sirtuin deacetylase (Rodríguez-Santana et al., 2023; Sun et al., 2019; Xia et al., 2023; Li et al., 2023). At the same time, cell metabolism and biorhythms interact, and specific metabolic pathways are key regulators of peripheral biorhythms. Oscillating metabolites can synchronize the biological clock, regulate transcription and chromatin accessibility (Lu et al., 2020; Mendez and Diaz-Munoz, 2018). Sensors with lower energy and nutrient status in cells disrupt the biological clock’s negative regulation of PERs and CRYs, changing the circadian rhythm (Um et al., 2007). Metformin is a well-tolerated hypoglycemic drug that can regulate many metabolic pathways. It indirectly regulates the expression of biorhythms (Alex et al., 2020; Miura et al., 2023; Albayrak et al., 2021). For example, metformin can regulate the PER2 expression by regulating CK1 and the expression of intracellular biorhythms in a tissue-dependent manner (Barnea et al., 2012). In this study, our transcriptomic results showed that PERs expression in GBM cell lines changed after metformin intervention. PER2 expression increased significantly, which confirmed that metformin can regulate the expression of certain biological rhythm genes in glioma cells. However, the precise underlying mechanism is elusive, which led us to explore it further.

Numerous studies have reported that metformin decreases morbidity and mortality in various malignancies, including prostate, endometrial, stomach, pancreatic, thyroid, breast, and colon cancer (Ng et al., 2020; Gallez et al., 2024; He et al., 2020; Lv and Guo, 2020; Papadakos et al., 2024; Marini et al., 2021; DePeralta et al., 2016; Shankaraiah et al., 2019). It can inhibit the proliferation and invasion of some tumors and has a sensitive effect on radiotherapy and/or chemotherapy (Papadakos et al., 2024; Coyle et al., 2016). Regarding this effect, the following mechanisms are involved, including inhibiting insulin-like growth factor-1 (IGF-1), activating adenosine monophosphate-activated protein kinase (AMPK) (Wang et al., 2019), inhibiting mammalian target rapamycin complex I (MTORC1), inhibiting nuclear factor-κB (NF-kB) (Li et al., 2020), and inhibiting oxidative phosphorylation in cancer cells. Metformin plays a chemo-preventative role in the development of hepatocellular carcinoma by inhibiting mTOR (Bhalla et al., 2012). It also regulates tumor glucose metabolism, especially the glycolytic pathway (Marini et al., 2021). However, its role in tumors varies across tissues. In this study, metformin affected GBM cell lines by regulating the SIRT2/G6PD signaling pathway and the PPP pathway in a PER2-dependent manner. The main products of PPP consist of reduced nicotinamide adenine dinucleotide (NADPH) and 5-phosphoribose (R-5P). Adequate NADPH and R-5P are necessary for cell growth and proliferation, especially for rapid-growing tumor cells (Ghergurovich et al., 2020). G6PD is at the heart of metabolic reprogramming in cancer (Yang et al., 2021). Previous studies have shown that G6PD plays an important role in tumor occurrence and development (Song et al., 2022). G6PD is usually upregulated in solid tumors and is associated with poor prognosis, making it a potential therapeutic target for cancer (Nagashio et al., 2019; Wang et al., 2021; Wang et al., 2020; Ramirez-Nava et al., 2021; Sun et al., 2021). Some studies have reported that G6PD is upregulated in gliomas, and it might be related to poor prognosis (Sun et al., 2021). Therefore, therapeutic strategies targeting G6PD may be important for treating GBM patients.

Intracellular biorhythms play an important role in tumor occurrence and development, and directly targeting biorhythm molecules to reset or rescue circadian genes is a promising strategy. Other potential research directions are regulating the biological rhythm oscillation in the tumor, restoring the cell’s rhythm to regulate and prevent tumors, and altering tumor cell proliferation. The results of this study provide us with important implications in this regard. Metformin upregulates the core clock gene PER2 in GBM cell lines, regulates the SIRT2/G6PD signaling pathway in a PER2-dependent manner, and has synergistic effects on the radiotherapy sensitivity of GBM cell lines. Researchers have reported that restoring the molecular clock has an inhibitory effect on neuroblastoma (NB). Reactivation of RORα could significantly improves the anti-tumor activity of etoposide and serve as a therapeutic strategy for MYCN-amplified NBs by blocking the dysregulation of molecular clock and cell metabolism mediated by MYCN (Moreno-Smith et al., 2021). Hence, we postulated that restoring the expression and oscillation of PER2 induced by metformin could effectively block GBM tumor growth and sensitizes GBM tumors to radiotherapy and or chemo-radiotherapy.

In this study, we found that metformin inhibited GBM cell lines in a concentration-dependent manner to some extent. In in vivo animal experiments, 200 mg/kg/day of metformin had a prominent tumor suppression and radiosensitive effect on xenograft GBM tumors in nude mice.

Several limitations should be noticed when translating these animal or pre-clinical findings into clinical treatment. The *in vitro* metformin doses used in studies are generally more concentrated than the reported physiological concentrations in systemic circulation, with one *in vitro* study using up to 100 mmol/L of metformin (Sun et al., 2016). Therapeutic doses of metformin in human systemic circulation, approximately 10–40 mmol/L may not be sufficient to inhibit tumor growth (He and Wondisford, 2015). Therefore, optimal strategy of delivering it to tumor cells may need to be explored. Song et al. designed a metformin–cisplatin nanoparticle activated by lasers and targets the ligand low-density lipoprotein receptor, which is specific to the hypoxic regions of HNSCC (Song et al., 2020; Zhao et al., 2019). Drug resistance in tumors is a critical issue in treatment, such as cisplatin resistance Wang et al., 2023). Metformin sensitizes the effect of cisplatin in cell cultures and *in vivo*. This novel drug delivery method can target xenograft tumors with high doses of cisplatin (10 mg/kg) combined with metformin (1 mg/kg) to overcome low systemic concentrations of metformin and reduce potential toxicities. Drug delivery methods incorporating metformin in microparticles or nanoparticles are currently being explored to overcome its low oral bioavailability and to target dose delivery for effective cancer treatment (Cetin and Sahin, 2015).

Sensitivity of metformin is greatly influenced by nutrient supply, which differs in in vivo and *in vitro* settings. Thus, it is challenging to predict the drug concentrations needed to achieve anti-tumor activity *in vivo* based on the existing *in vivo* research data from animal experiments. So in future clinical applications, determining what is the ideal drug concentration, how and when to give to tumor regions without causing serious side effects will be challenging and the most important aspects of our future explorations.

In sum, circadian clock genes PER2 plays a pivotal role during GBM tumor onset and development. A therapeutic strategy based on the PER2/SIRT2/G6PD pathway might be a novel, effective, and fascinating strategy for treating GBM patients. As a widely used hypoglycemic drug in clinical, further exploration of the mechanism of action is expected to provide new breakthroughs in regulating circadian rhythms *in vivo* and even within tumors. The mechanisms of metabolic reprogramming in tumors are complex (Xie et al., 2024), in-depth research on the mechanisms of metformin in tumors and its application in targeted control after administration can be conducted with the hope that metformin will play a greater role in chronotherapy on tumors in clinical practice.

## 4 Materials and methods

### 4.1 Cell culture

Human GBM cell lines U87 and U251 and astrocytoma cell lines HA1800 were purchased from the Cell Bank Type Culture Collection of the Chinese Academy of Sciences and were frozen in liquid nitrogen. The cell lines were authenticated with short tandem repeat (STR) profiling by a third-party company in 2021. GBM cell lines were cultured in DMEM (Biological Industries; Israel) containing 10% fetal bovine serum (Biological Industries; Israel) and 1% penicillin and streptomycin at 37°C in a humidified atmosphere of 5% CO_2_ (v/v). HA1800 cell lines were cultured in specialized astrocyte medium. *PER2* knock-down and overexpression lentivirus with green fluorescence protein (GFP) were purchased from Shanghai GeneChem Co., Ltd. (Shanghai, China), and the extent of GFP expression was observed with a fluorescence microscope. The trans-infection efficiency of U87 and U251 at 72 h following lentiviral transduction was confirmed via real time qPCR and Western blotting (WB) analysis.

### 4.2 Cell counting Kit-8 (CCK8) assay

U87/U251 cells (1*10^5^) in the logarithmic growth stage were selected from a 5% CO_2_ cell culture at 37°C and treated with metformin at escalating concentrations for 24 h. The cells were washed twice with PBS at room temperature, 110 μL of medium containing CCK8 was added (the ratio of medium to CCK8 was 10:1), and incubation continued for 2 h. Then we detected the absorbance at 450 nm and record the results.

### 4.3 G6PDH enzyme activity assay

U87/U251 cell lines in good state were treated with metformin for 24 h. Then, the levels of G6PDH enzymatic activity were detected using glucose-6-phosphate dehydrogenase (G6PDH) activity assay kit (Cat^#^BC0265, Beijing Solarbio Science & Technology Co. Ltd). The operating procedure is carried out according to the instructions. G6PDH enzyme activity is proportionally related to and reflected by the concentration of NADPH which is detected at 340 nm absorbance.

### 4.4 Intracellular NADPH levels detection and NADP^+^/NADPH ratio measurement

The intracellular NADPH levels of U87/U251 cell lines treated with/without metformin were measured using the NADP/NADPH assay kit (Cat^#^ab65349, Abcam, UK). The operation procedure is carried out according to the kit instructions. Intracellular NADPH levels can be quantified using a plate reader at OD 450 nm.

### 4.5 ROS and JC-1 measurement

U87/U251 cell lines following treatment were detected using ROS Asay Kit (Cat^#^S0033S, Beyotime Biotechnology, China) and JC-1 levels were quantified using JC-1 Asay Kit (Cat^#^C2006, Beyotime Biotechnology, China). The cell lines were detected via flow cytometry.

### 4.6 Cell cycle distribution and apoptosis analysis

U87/U251 cell lines following treatment were detected using flow cytometry. The cell cycle distributions were detected with Cell Cycle and Apoptosis Analysis Kit (Cat^#^C1052, Beyotime Biotechnology, Shanghai). The apoptosis analyses were detected stained with Annexin V-FITC (Cat^#^BB-4101–2, BestBio Biotechnology, Shanghai) and Annexin V Alexa Fluor 660/7-AAD (Cat^#^BB-41038, BestBio Biotechnology, Shanghai) and analyzed in a Cytomic FC500 Flow Cytometry System (Beckman Coulter, Krefeld, Germany).

### 4.7 Transcriptomic

Total RNA from U87 cell lines following treatment was extracted using TRIzol (Invitrogen; Thermo Fisher Scientific, Inc., United States) and treated with DEPC water. RNA Seq was performed by Genechem Technology Co., Ltd. (Shanghai, China). Differential expression analysis was conducted with DEGseq2 software. Genes with adjusted *P* values (*P* adjust) of ≤0.05 were considered DEGs. KEGG pathway and GO analyses of DEGs were performed with cluster profiler. Genes with an adjusted *P*-value < 0.05 found by DESeq2 were assigned as differential expressed.

### 4.8 Proteomic

Total protein from U87 cell lines treated with metformin for24 h was extracted for the following experiment. Proteomic was performed by Genechem Technology Co., Ltd. (Shanghai, China). Protein with fold change>1.2 and p value (Student’s t-test) < 0.05 were considered to be differential expressed proteins. The results were analyzed using the UniProt database (Uniprot_HomoSapiens_20387_20210928_9606_swissprot).

### 4.9 Western blot tests

A whole-protein extraction kit was used to extract the proteins, and the protein content was assessed using a BCA kit (KeyGEN Biotech, Jiangsu, China) The protein samples underwent sodium dodecyl sulfate polyacrylamide gel electrophoresis (SDS-PAGE) in equal quantities (70 V, 30 min, switch to 120 V, 90 min), followed by polyvinylidene difluoride (PVDF) membrane transfer at 300 mA for 130 min and blocking in 5% BSA for 1 h. Next, the antibodies were incubated at 4°C for the entire night on a shaking bed. The next day, the membrane was treated with second antibody for 1 hour at room temperature on a shaking bed. After the Western blot tests, chemiluminescence images and ImageJ were used to evaluate the data. The antibodies used in the study are as follows: anti-G6PD antibody (Cat^#^ab133525; Abcam, UK); anti-PER2 antibody (Cat^#^ab179813; Abcam, UK); anti-SIRT2 antibody (Cat^#^ab211033; Abcam, UK).

### 4.10 Real-time quantitative polymerase chain reaction experiments (RT-qPCR)

Total RNA was extracted from the GBM cell lines. The PrimeScript™ RT reagent Kit (Cat#RR037A, TaKaRa, China) was used to reverse-transcribe the RNA into cDNA, and RT-qPCR was conducted using FastFire qPCR premix. The primer sequences are list below (Table 1). The primers were obtained from Shanghai Bioengineering Company. iQ5 (Bio Rad Laboratories, Inc. Hercules, CA, United States) was employed for PCR amplification. The 2^−ΔΔCT^ technique determined the relative expression of the genes. Three iterations of these experiments were conducted.

### 4.11 *In vivo* experiment

An animal xenograft tumor formation experiment was conducted on nude mice. The experiments were carried out at animal center with the approval of the Ningxia Medical University Experimental Animals Center IACUC. U87 cell lines in a good growth state at a concentration of 1 × 10^7^ cells/mL were inoculated into the subcutaneous skin of the right buttock of total 40 nude mice. The nude mice were divided into four group randomly and were given metformin at the dosage of 100 mg/kg·d, 200 mg/kg·d, 300 mg/kg·d, or saline via intragastric gavage administration respectively. The animal weight and tumor growth were recorded. External beam radiotherapy of 6 MV X-Ray was delivered when the xenograft tumor volume reached 0.5 cm^3^ with total dose of 18Gy/6Gy/3fractions. After radiotherapy, the tumor and animal organs were dissected under inhalation anesthesia. The tumor mass was measured, the tumor inhibition rate was calculated, and the synergistic effect was evaluated using the Jung-average formula. HE, IHC and immunofluorescence detection were performed.

### 4.12 Immunohistochemistry (IHC)

IHC was performed using the standard SP method. The antibodies used were as follows: anti-G6PD antibody (Cat^#^ab133525; Abcam, UK); anti-PER2 antibody (Cat^#^ab179813; Abcam, UK); anti-SIRT2 antibody (Cat^#^ab211033; Abcam, UK); anti-Bcl-2 antibody (Cat^#^WL01556; Wanleibio, China); anti-Bax antibody (Cat^#^WL01637; Wanleibio, China); anti-ROS antibody (Cat^#^ab189925; Abcam, UK). A Leica DM6 fluorescent microscope (Leica camera AG, Wetzlar, Germany) was used to capture images, and ImageJ was used for analyses.

### 4.13 TUNEL staining

The paraffin embedding and dewaxing processes followed the procedure described above. Based on the kit’s instructions (Cat^#^WLA030, Wanleibio, China; Cat^#^ D106471, Aladdin, China), the samples were first treated with a proteinase working solution. A Leica DM6 fluorescent microscope (Leica Camera AG, Wetzlar, Germany) was used to capture images, and ImageJ was used for analyses.

### 4.14 Statistical analysis

Prism9.5.1 was used to perform statistical analyses on all of the data. Data are presented as the mean ± SD. Two independent groups were compared using Student’s t-test, and the chi-square test was used for variance analysis. One-way analysis of variance (ANOVA) was used to compare data among more than two groups. P < 0.05 was considered to indicate a significant difference. The results were considered statistically significant at *P* < 0.05 (*P < 0.05, **P < 0.01, ***P < 0.001, and ****P < 0.0001).

## 5 Conclusion

Overall, our results suggest that metformin could inhibit the cell growth, proliferation, invasion, and migration of GBM cell lines. Metformin regulates GBM cell lines through the circadian gene *PER2* and the SIRT2/G6PD signaling pathway in a *PER2*-dependent manner, as well as through synergies with the radiotherapy sensitivity of glioma cells. Our findings can be summarized in three points: First, pharmacologically regulating the biological clock will be a research direction and goal in future joint research. Metformin could regulate the expression of biological rhythms of GBM cell at molecular level. That makes it possible to rescue the misalignment of biological rhythms in tumors through the drug re-purposing. And this may be a great progress of chronotherapy toward its clinical application. Second, we innovatively investigated the effect of metformin on the PPP pathway. Metformin can inhibit the PPP flux on GBM cell lines and reverse the metabolic reprogramming. Third, the key rate-limiting enzymes of the PPP pathway present rhythmic expression, so this pathway can be modulated by circadian genes. This indicates that further exploring these enzymes could provide potential valuable and novel information on metformin’s impact on cancer stem cells and tumor micro-environment.

## Data Availability

The datasets presented in this study can be found in online repositories. The names of the repository/repositories and accession number(s) can be found in the article/Supplementary Material.
