# Supplementary material for: Metformin upregulates circadian gene PER2 to inhibit growth and enhance the sensitivity of glioblastoma cell lines to radiotherapy via SIRT2/G6PD pathway
Source: Front Pharmacol. 2025 Mar 17;16:1563865. doi: 10.3389/fphar.2025.1563865 (PMC11955593; doi:10.3389/fphar.2025.1563865)
Supplement: Supplementary file 1 [file DataSheet1.docx]

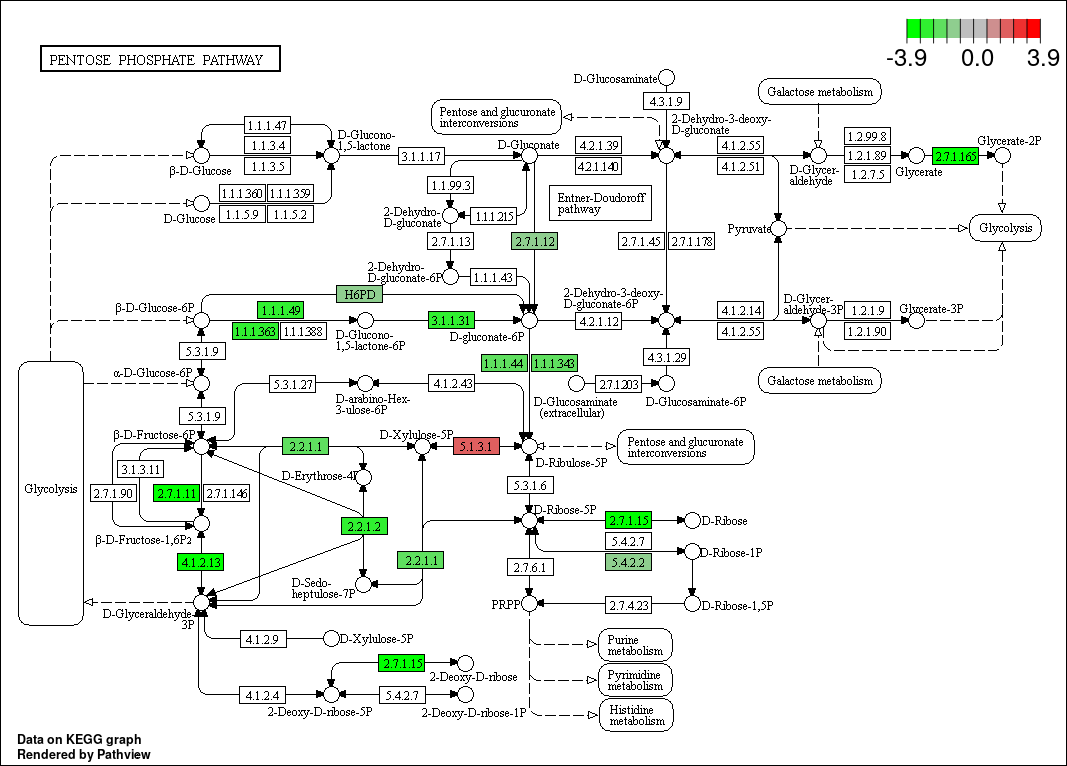
 **Figure 1.** KEGG map (hsa00030). The boxes represent genes, and the circles represent metabolites. Color gradients are used to show specific expressions.

**Table 1**. **Co-expression pathways and the proteins and genes involved.**

| **Kegg** | **Pathway name** | **Gene** | **protein** |
| --- | --- | --- | --- |
| hsa00030 | Pentose phosphate pathway(PPP) | TALDO1, PGD, G6PD, TKT, PFKL,  PGLS, GLYCTK, PFKP, H6PD, PGM1  ALDOC, AC093512.2, RBKS, IDNK | G6PD |


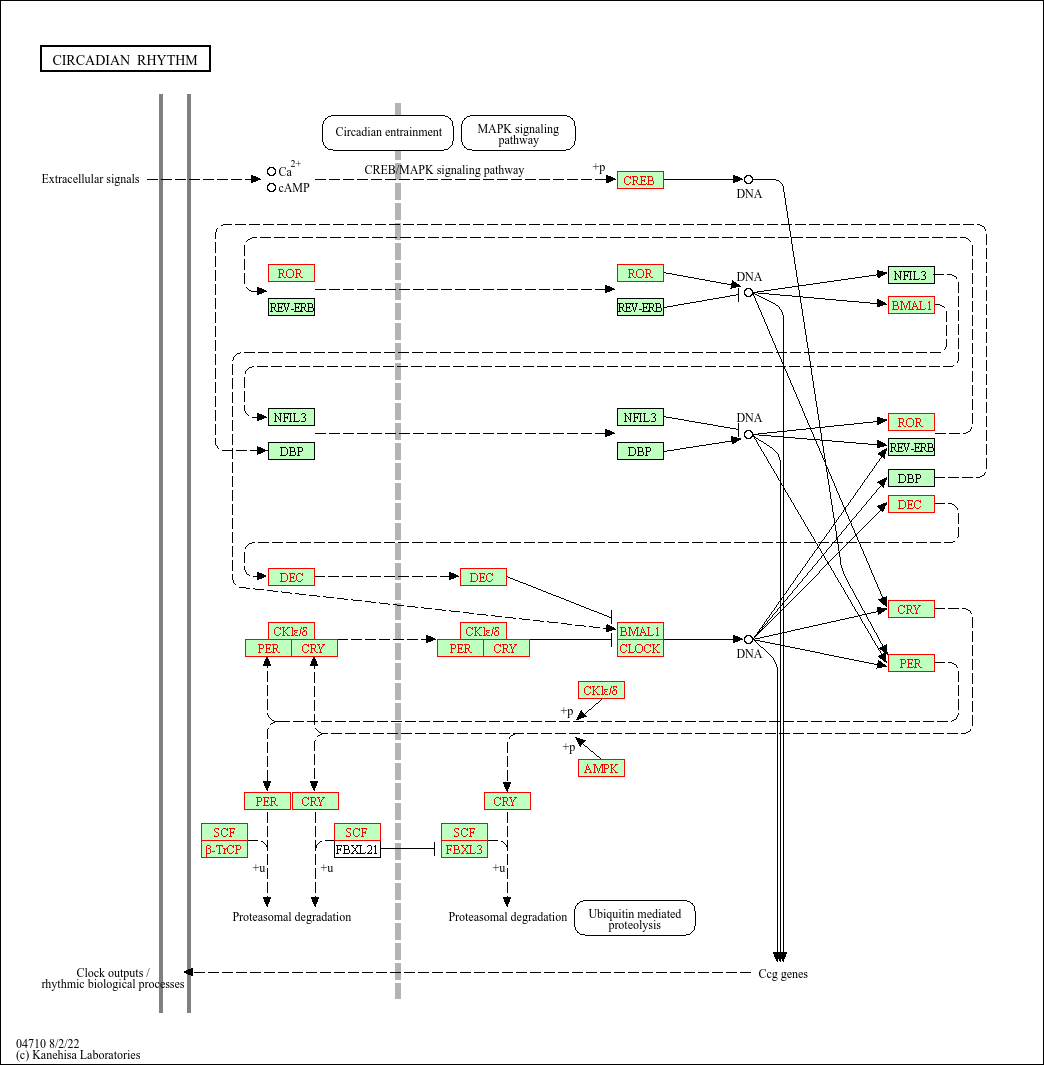


**Figure 2** KEGG map (hsa04710). The boxes represent genes /proteins, and the circles represent metabolites. Color gradients are used to show specific expressions.

**Table 2. Co-expression pathways and the proteins and genes involved.**

| **Kegg** | **Pathway name** | **Gene** |
| --- | --- | --- |
| hsa04710 | Circadian rhythm | PRKAB2, CUL1, PER2, CRY2, CSNK1E, FBXL3, PRKAA1, BHLHE41, PRKAG2, PER3, RORA, SKP1, CLOCK,CREB1,CRY1,BTRC,FBXW11,RBX1,PRKAG1 |


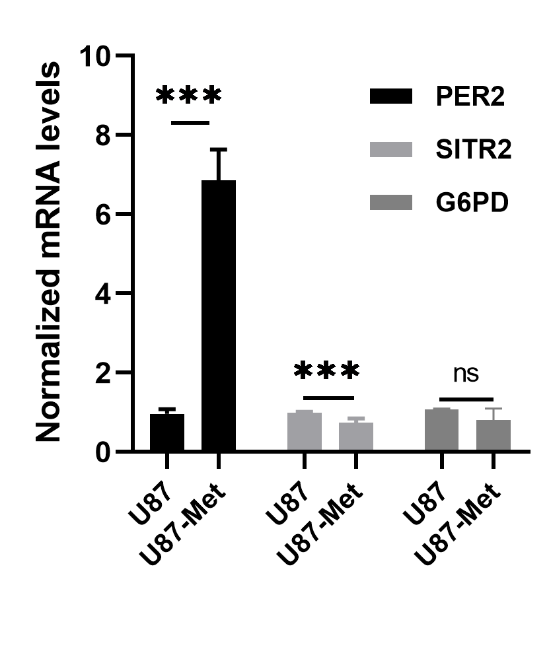

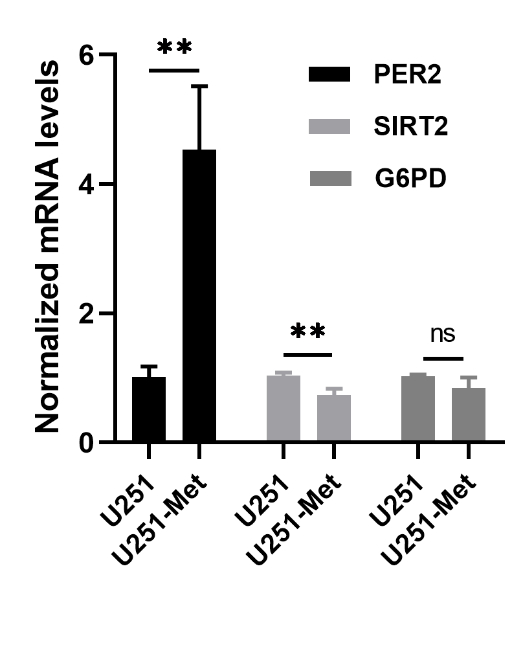
 **Figure 3**. Validation of mRNA levels of *PER2*, *SIRT2*, *G6PD* treated with metformin in GBM cell lines.
